# Potential association of the prognostic index and survival in patients with p16-positive oropharyngeal squamous cell carcinoma

**DOI:** 10.1007/s00508-021-01885-0

**Published:** 2021-06-18

**Authors:** Faris F. Brkic, Christina Mayer, Gerold Besser, Gabriela Altorjai, Harald Herrmann, Gregor Heiduschka, Georg Haymerle, Lorenz Kadletz-Wanke

**Affiliations:** 1 Department of Otorhinolaryngology, Head and Neck Surgery, Medical University of Vienna, Währinger Gürtel 18-20, 1090 Vienna, Austria; 2 Department of Radiation Oncology, Medical University of Vienna, Währinger Gürtel 18–20, 1090 Vienna, Austria

**Keywords:** Squamous cell carcinoma, Head and neck, HPV, Outcome, Prognostic marker

## Abstract

**Background:**

The aim was to assess the prognostic value of the newly proposed prognostic index (PI) in patients with p16-positive oropharyngeal squamous cell carcinoma.

**Methods:**

Patients treated with primary surgery from 2012 to 2019 with available preoperative (0–2 days) values of C‑reactive protein and white blood cell counts needed for calculation of the PI, were included. Main outcome measures were overall survival (OS) and disease-free survival (DFS). The PI was dichotomized into low (PI = 0) and high (PI ≥ 1).

**Results:**

In this study 36 patients were included. Average overall (OS) and disease-free survival (DFS) were 3.3 years (range 0.2–12.3 years) and 2.8 years (0.0–9.8 years), respectively. The overall mortality was 16.7% (*n* = 6) and a recurrent disease was observed in 30.6% of patients (*n* = 11). Low PI was associated with better overall survival (mean OS 10.1 ± 1.4 years, 95% confidence interval, CI 7.3–12.9 years vs. 1.9 ± 0.4, 95% CI 1.3–2.6 years, *p* < 0.01; mean DFS 8.5 ± 0.7 years, 95% CI 7.1–9.6 years vs. 1.0 ± 0.3 years, 95% CI 0.5–1.5 years, *p* < 0.01).

**Conclusion:**

The PI might be an easily obtainable outcome prognosticator in p16-positive oropharyngeal squamous cell carcinoma patients. Analyzing routinely obtained blood samples can contribute to identifying high-risk patients.

## Introduction

About 500,000 new cases of head and neck squamous cell carcinoma (HNSCC) are diagnosed annually and it is currently listed as the 6th most common malignancy in the world [1]. In the western world, this cancer entity has shown a gradual decrease of incidence numbers, which is mainly associated with lower rates of tobacco smokers. On the contrary, the incidence of a subgroup of HNSCC, namely oropharyngeal squamous cell carcinoma (OPSCC), is continuously increasing [2]. The human papilloma virus (HPV) may be the main reason for the incidence rise of this cancer type [3].

An upregulation of the p16 protein, which is mediated by the HPV virus, can be detected by immunohistochemical staining. A p16 staining is the most widely used method for determining the HPV status as the concordance rates are about 95% [4]. An HPV-associated OPSCC is considered to have a completely different tumor biology as compared to HPV-negative HNSCC. Most importantly, it is usually linked to good clinical outcome with higher survival rates [5]. Furthermore, it has been noted that HPV-positive cases of OPSCC have different predictors of outcome than HPV-negative cases [6]. Traditional prognosticators of worse survival, such as perineural invasion, extracapsular spread or positive surgical margins, do not seem to have the same prognostic value in HPV-positive OPSCC [6]. Despite excellent survival rates in even locoregionally advanced cases, there is still a subset of patients that will die of this disease. Thus, markers still need to be identified, which help us select for high-risk cases in a cohort of patients with otherwise favorable outcome and may benefit from treatment intensification.

The principle that carcinogenesis and inflammation are inextricably linked is well established. It has been hypothesized that both systemic and in the tumor-microenvironment, inflammation plays an important role in the promotion of carcinogenesis [7]. Recently, it was proposed that the complex interplay between systemic inflammation and the tumor microenvironment is reflected by inflammatory biomarkers. From there, the idea emerged that these easily obtainable inflammation markers have the potential to predict outcome in cancer patients [8]. Up to now, different inflammatory markers were shown to be associated with survival in head and neck cancer patients. These included absolute leukocyte, lymphocyte or platelet counts, C‑reactive protein (CRP) and combinations of these, such as neutrophil-to-lymphocyte ratio (NLR) or platelet-to-lymphocyte ratio [9–12].

Recently, the prognostic index (PI) was introduced as a tool to predict prognosis in cancer patients. It is determined by combining the values of C‑reactive protein (CRP) and white blood cells (WBC). The PI has shown a significant association with survival in patients with small cell lung cancer [13] as well as in surgically treated patients with pancreatic ductal adenocarcinoma [14]. To date, its impact on outcome in head and neck cancer patients, and particularly in HPV-associated OPSCC has not been evaluated.

Therefore, the aim of this study was to examine if the PI has a prognostic value on the outcome in patients with p16-positive OPSCC.

## Material and methods

This retrospective cohort study was conducted at a tertiary academic medical referral center (Department of Otorhinolaryngology, Head and Neck Surgery, Medical University of Vienna, Austria). Inclusion criteria were classified as newly diagnosed p16-positive OPSCC, treated with primary surgery from 2012 to 2019, and available preoperative (0–2 days) values of CRP and WBC. Primary outcome measure was the impact of the PI on overall (OS) and disease-free survival (DFS).

### P16 staining

The p16 status was determined using immunohistochemistry according to protocols provided by Ventana (CINtec p16 Histology kit, Roche Tissue Diagnostics, Ventana, Tucson, AZ, USA). Positive staining was defined as block staining with strong nuclear and cytoplasmic expression in a continuous segment of cells (at least 10 cells).

### Prognostic index

The PI is determined by combining the CRP and WBC values as shown (Table 1). For our analysis, blood samples collected during routine preoperative evaluation were used. Patients were categorized into PI low (PI = 0) and PI high (PI ≥ 1) based on recommendations of two previously published studies [13, 14].

**Table 1:** Dichotomization into high and low according to CRP and WBC values

Prognostic index	CRP (mg/L)	WBC (G/l)	*n* (%)	Prognostic index	*n* (%)
0	≤ 10	≤ 11	27 (75.0)	Low	27 (75.0)
1	≤ 10	> 11	8 (22.2)	High	9 (25.0)
1	> 10	≤ 11			
2	> 10	> 11	1 (2.8)		

*CRP* C-reactive protein, *WBC* white-blood cell count

### Statistics

Statistical analysis was performed using the Statistical Program of Social Sciences (SPSS Version 23.0, IBM Corp., Armonk, NY, USA). Statistical significance was set at 0.05, two-tailed. Kaplan-Meier curves were computed to analyze survival rates. Log-rank test was used to evaluate for statistical significance. Patients were dichotomized into low PI (0) and the high PI (PI ≥ 1) group. In order to show the variability of data, 95% confidence intervals (CI) were reported. Figures were created using GraphPad Prism version 9.0.2 for macOS (GraphPad Software, La Jolla, CA, USA, www.graphpad.com).

## Results

### Basic clinical characteristics

A total of 53 patients underwent primary surgical treatment for HPV-positive OPSCC at our institution during the study period. Preoperative (0–2 days) WBC and CRP values were available in 36 patients and were used for subsequent analysis.

The mean age of the cohort was 66.0 years (range 37.0–85.4 years; median 68.2 years). Of the patients 24 (66.6%) were male and 12 (33.3%) were female. Small tumors were observed in the majority of patients. T1 and T2 tumors were observed in 12 (33.3%) and 18 (50.0%) patients, respectively, 3 patients (8.3%) were diagnosed with a T3 primary and 2 (5.5%) with a T4 tumor. Positive local lymph nodes (*N* +) were present in the plethora of all patients (*n* = 35; 97.2%). None of the patients had distant metastases at the time of surgery. A total of 24 patients (66.6%) had a moderately differentiated (G2) and 12 (33.3%) a G3 carcinoma. Postoperative radiotherapy (PORT) was performed in 28 patients (77.8%) with 7 patients (19.4%) receiving a concurrent chemotherapy. Mean OS and DFS of all patients were 3.3 years (range 0.2–12.3 years) and 2.8 years (0.0–9.8 years), respectively. The overall mortality was 16.7% (*n* = 6) and 30.6% of patients (*n* = 11) suffered from recurrent disease.

### Prognostic index

Mean values of CRP and WBC were 5.1 mg/L (range 0.2–35.2 mg/L) and 7.4 G/L (range 4.1–16.1 G/L), respectively. Based on these parameters, the PI was calculated. As proposed by Gruber et al. [14] patients were stratified into low PI (PI 0) and high PI (PI ≥ 1) groups (Table 1). Thus, a low PI was observed in 27 patients and a high PI in 9 patients.

Tumor and patient characteristics according to the PI value are presented in Table 2. In summary, no significant differences were observed between the two patient groups regarding demographic or tumor characteristics. On average, patients with a low PI were slightly older (median 69.2 years vs. 62.9 years). A T1/T2 stage was observed in 88.9% patients with a low PI; on the contrary, 66.6% of patients with a high PI presented in a T1/T2 stage. Surgical margins were positive in 40.1% of patients with a low PI and in 33.3% of patients in the high PI group.

**Table 2:** Tumor and patient characteristics according to the PI

Factors	Study cohort	Low PI (0)	High PI (1 or 2)
Study cohort	–	***n*** **=** **27 (75%)**	***n*** **=** **9 (25%)**
**Sex**			
Male, *n* (%)	24 (66.6)	19 (70.4)	5 (55.6)
Female, *n* (%)	12 (33.3)	8 (29.6)	4 (44.4)
**Age**			
Median (range), years	68.2 (37.0–85.4)	69.2 (37.0–85.4)	62.9 (52.0–77.0)
**T Stage**			
T1, *n* (%)	12 (33.3)	10 (37.0)	2 (22.2)
T2, *n* (%)	18 (50.0)	14 (51.9)	4 (44.4)
T3, *n* (%)	3 (8.3)	2 (7.4)	1 (11.1)
T4, *n* (%)	2 (5.5)	1 (3.7)	2 (22.2)
**N Stage**			
N0, *n* (%)	35 (97.2)	26 (96.2)	9 (100.0)
N1, *n* (%)	1 (2.8)	1 (3.8)	0 (0.0)
N2, *n* (%)	0 (0.0)	0 (0.0)	0 (0.0)
**Grading**			
G1, *n* (%)	0 (0.0)	0 (0.0)	0 (0.0)
G2, *n* (%)	24 (66.6)	18 (66.6)	6 (66.6)
G3, *n* (%)	12 (33.3)	9 (33.3)	3 (33.3)
**PORT**			
Yes, *n* (%)	28 (77.8)	21 (77.8)	7 (77.8)
No, *n* (%)	8 (22.2)	6 (22.2)	2 (22.2)
**Mortality**			
*n* (%)	6 (16.7)	2 (7.4)	4 (44.4)
**Recurrence**			
*n* (%)	11 (30.6)	5 (18.5)	6 (66.7)
**Surgical margin**			
Positive, *n* (%)	14 (38.9)	11 (40.1)	3 (33.3)
Negative, *n* (%)	22 (61.1)	16 (59.9)	6 (66.6)

*PI* prognostic index, *PORT* postoperative radiotherapy

### Survival analysis

**Fig. 1:**
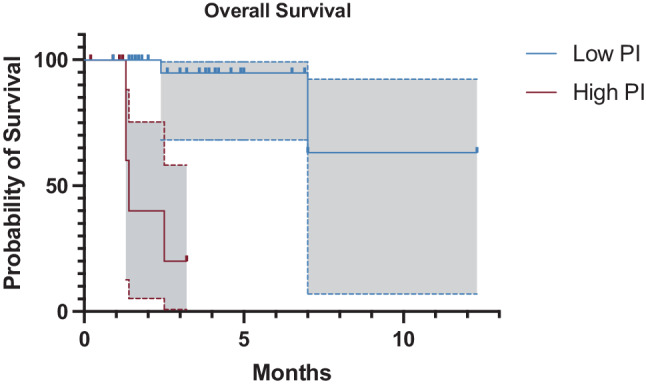
Kaplan-Meier curve comparing overall survival between patients with low and high preoperative PI values (*p* < 0.01). *Grey areas bordered by dotted lines* represent the respective 95% confidence interval. *PI* prognostic index

**Fig. 2:**
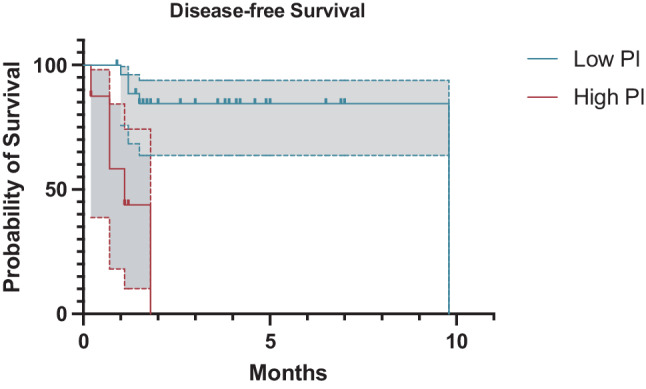
Kaplan-Meier curve comparing disease-free survival between patients with low and high preoperative PI values (*p* < 0.01). *Grey areas bordered by dotted lines* represent the respective 95% confidence interval. *PI* prognostic index

OS and DFS curves for patients in dependence of their PI category are displayed in Figs. 1 and 2. The OS and DFS for the whole cohort were 8.9 ± 1.3 years (95% CI 6.3–11.5) and 7.1 ± 0.8 (95% CI 5.6–8.6), respectively. Statistically significant better OS and DFS was observed for patients with low preoperative PI; Survival time: Mean OS 10.1 ± 1.4 years (95% CI 7.3–12.9) vs. 1.9 ± 0.4 years (95% CI 1.3–2.6), *p* < 0.01; Mean DFS 8.5 ± 0.7 years (95% CI 7.1–9.6) vs. 1.0 ± 0.3 years (95% CI 0.5–1.5), *p* < 0.01.

## Discussion

This study assessed the prognostic value of the PI in patients with HPV-positive OPSCC. To the best of our knowledge this is the first evaluation of the prognostic value of this marker on survival in any of head and neck cancer. We were able to show that low PI was highly significantly associated with better OS and DFS in our cohort of 36 patients with p16-positive OPSCC undergoing surgical resection.

The PI incorporates the combination of CRP and WBC values, and was recently introduced as a simple marker in cancer patients with a potential prognostic value [13, 15]. Our results correspond to those reported in other studies examining other cancer entities. Kasymjanova et al. showed that a high PI is significantly associated with poor outcome in patients with advanced non-small cell lung cancer [13]^.^ Another group described that the prognostic power of this marker might be useful in patients with pancreatic ductal adenocarcinoma undergoing surgery [14]. They reported that high PI predicts worse disease-specific survival.

Prognostic values of other inflammation markers have been already assessed in HNSCC. It has been shown that these easily obtainable biomarkers can contribute to identifying high-risk patients. They are based on the hypothesis that the inflammation is a cancer hallmark and one of the main promotors of carcinogenesis [15]. Moreover, it has been proposed that both systemic as well as inflammation in the tumor microenvironment play an important role in carcinogenesis [7]. The complex interaction between systemic and tumor-specific inflammation may be reflected by different inflammation biomarkers [8]. Their prognostic values have been reported for different head and neck cancer entities. For example, the NLR was shown to be a strong survival prognosticator in laryngeal and HPV-negative squamous cell carcinoma of unknown primary [16, 17]^.^ Furthermore, two studies assessing inflammation in OSCC patients noted that high pretreatment CRP and WBC correlated with worse outcomes [15, 18].

Data on prognostic markers in HPV-positive OPSCC are generally scarce [6]. Regarding inflammation markers, Johnson-Obaseki et al. [10] found no association of pretreatment CRP values with outcome in HPV-positive OPSCC. Another study was able to show that lower NLR was associated with lower rates of regional lymph node spread; however, this cohort included only a limited number of patients [19]. One study group was able to show that high pretreatment NLR associates with poor DFS in HPV-positive OPSCC [20]. Recently, it has been noted that PD-L1 (Programmed cell death 1 ligand 1) positive immune cells are a possible marker for HPV-positive OPSCC patients with an excellent outcome [21]. This study group proposed that these patients could be suitable for trials evaluating de-escalation of treatment in HPV-positive OPSCC.

Currently, treatment de-escalation for HPV-associated OPSCC is under emerging discussion [6]. Firstly, several recent phase III studies have been designed in order to assess the use of cetuximab instead of cisplatin with concurrent standard dose radiotherapy. It was shown that this de-escalation approach is not suitable for HPV-associated OPSCC patients. Furthermore, some investigators proposed the use of induction chemotherapy in order to identify future responders for subsequent radiotherapy. Reduction of radiation dose might lower the rate of therapy-associated comorbidities while being a safe therapy alternative in selected cases [22]. Nevertheless, these attempts are limited by the fact that some patients with HPV-positive OPSCC are associated with a noticeably worse course of disease [23]. Up to now, there are no markers in routine use that help clinicians to select for high-risk HPV-positive patients that may need more aggressive therapy regimens. This study may provide additional information regarding this issue and could be the basis for future studies.

The main limitation of the study is the limited number of patients with available data on pretreatment values of CRP and WBC. Moreover, due to the retrospective study character, some follow-up data could have possibly been missed (e.g., follow-up in another center). Lastly, *p*-values were not adjusted for multiple testing and should be interpreted only exploratorily.

## Conclusion

The PI might serve as a strong prognostic tool for predicting OS and DFS in p16-positive OPSCC patients treated with a primary surgical resection. The pretherapeutic values of CRP and WBC are easily obtainable for patients that undergo surgical resection. Thus, determining the PI can be a convenient tool for establishing a prognostic profile for each patient, which may contribute to identifying high-risk patients; however, due to the small cohort further studies with larger sample sizes are needed for validation of PI in p16-positive OPSCC.
