# Correction to: Potential association of the prognostic index and survival in patients with p16-positive oropharyngeal squamous cell carcinoma

**DOI:** 10.1007/s00508-021-01913-z

**Published:** 2021-08-03

**Authors:** Faris F. Brkic, Christina Mayer, Gerold Besser, Gabriela Altorjai, Harald Herrmann, Gregor Heiduschka, Georg Haymerle, Lorenz Kadletz-Wanke

**Affiliations:** 1 Department of Otorhinolaryngology, Head and Neck Surgery, Medical University of Vienna, Währinger Gürtel 18-20, 1090 Vienna, Austria; 2 Department of Radiation Oncology, Medical University of Vienna, Währinger Gürtel 18–20, 1090 Vienna, Austria

**Correction to:**

**Wien Klin Wochenschr 2021**

10.1007/s00508-021-01885-0

Unfortunately, the original version of this ar cle contained several mistakes. Lorenz Kadletz-Wanke was wrongly affiliated with the Department of Radiooncology and -therapy, Medical University of Vienna, Vienna, Austria. In addition, there was a mistake in the summary and results section. It should be 30.6% recurrence rate instead of 30.1%. The original article has been corrected. Open access funding provided by Medical University of Vienna.

